# Ocular biometric measurements in cataract surgery candidates in Portugal

**DOI:** 10.1371/journal.pone.0184837

**Published:** 2017-10-05

**Authors:** Tiago B. Ferreira, Kenneth J. Hoffer, Filomena Ribeiro, Paulo Ribeiro, João G. O’Neill

**Affiliations:** 1 Department of Refractive Surgery, Hospital da Luz, Lisbon, Portugal; 2 NOVA Medical School, Lisbon, Portugal; 3 Stein Eye Institute, University of California, Los Angeles, CA, United States of America; 4 St. Mary’s Eye Center, Santa Monica, CA, United States of America; 5 Faculdade de Ciências e Tecnologia, Lisbon, Portugal; Massachusetts Eye & Ear Infirmary, Harvard Medical School, UNITED STATES

## Abstract

**Objective:**

Describe the ocular biometric parameters and their associations in a population of cataract surgery candidates.

**Methods:**

A cross-sectional study of 13,012 eyes of 6,506 patients was performed. Biometric parameters of the eyes were measured by optical low-coherence reflectometry. The axial length (AL), mean keratometry (K) and astigmatism, anterior chamber depth (ACD) (epithelium to lens), lens thickness (LT), and Corneal Diameter (CD) were evaluated.

**Results:**

The mean age was 69 ± 10 years (44–99 years). Mean AL, Km, and ACD were 23.87 ± 1.55 mm (19.8–31.92 mm), 43.91 ± 1.71 D (40.61–51.14 D), and 3.25 ± 0.44 mm (2.04–5.28 mm), respectively. The mean LT was 4.32 ± 0.49 mm (2.73–5.77 mm) and the mean CD was 12.02 ± 0.46 mm (10.50–14.15 mm). The mean corneal astigmatism was 1.08 ± 0.84 D (0.00–7.58 D) and 43.5% of eyes had astigmatism ≥ 1.00 D. Male patients had longer AL and ACDs (p < .001) and flatter corneas (p < .001). In regression models considering age, gender, Km, ACD, LT, and CD, a longer AL was associated with being male and having higher ACD, LT and CD.

**Conclusions:**

These data represent normative biometric values for the Portuguese population. The greatest predictor of ocular biometrics was gender. There was no significant correlation between age and AL, ACD, or Km. These results may be relevant in the evaluation of refractive error and in the calculation of intraocular lens power.

## Introduction

With the increase in life expectancy of populations, there has been a progressive increase in the volume of cataract surgery performed worldwide, and it is the most common elective surgery in many countries. The introduction of less invasive techniques, new intraocular lenses (IOLs), and the achievement of more predictable refractive outcomes have been accompanied by an increase in patients' expectations of good visual outcome without the use of spectacles. Accurate biometric measurements are therefore essential. Knowledge of these measures is fundamental for obtaining precise calculations for the IOL power, which is primarily based on formulas derived from normative ocular biometric parameters.

It is known that ocular biometric parameters such as axial length (AL), corneal power (K), and anterior chamber depth (ACD) (corneal epithelium to anterior lens) vary with gender, age, and ethnicity, and hence are different among different populations. [1–6] Although there are many studies that describe these mean parameters in the European Caucasian population, there has been little attention to those studies carried out in Asian, Black and Hispanic populations. [1, 2] In addition, many of the published studies were conducted using contact applanation ultrasound biometry, a method limited by several measurement errors limiting its use prior to cataract surgery, particularly when premium lenses are implanted. It is known that optical biometry offers several advantages even over immersion ultrasound biometry, including its non-contact method, greater reproducibility and accuracy, and application in particular cases such as posterior staphyloma and eyes filled with silicone oil. [7, 8] Published studies of ocular biometric parameters using optical biometry are scarce, and this technology is constantly evolving and allows the evaluation of new parameters, such as measurement of the crystalline lens thickness (LT). Among various optical biometry devices available, Lenstar (Haag-Streit AG, Koeniz, Switzerland) has proved to be highly accurate in biometry measurements. [9]

There has not before been a large study of biometric values for the Portuguese population. The objective of the present study is to characterize the ocular biometric parameters and their associations in a population of cataract surgery candidates in Portugal.

## Material and methods

A retrospective study of 13,012 eyes of 6,506 patients who underwent cataract surgery was performed at the Hospital da Luz in Lisbon. The study was conducted in accordance with the principles of the declaration of Helsinki, and was approved by the center’s ethics committee (Hospital da Luz ethics committee). All patients provided written informed consent.

Ocular biometric parameters, including AL, mean corneal power K and astigmatism, ACD, LT, and corneal diameter (CD) were studied by optical low-coherence reflectometry using the Lenstar LS900 (Haag-Streit AG, Köniz, Switzerland). Examinations that yielded poor quality or uncertain results were excluded as were those of patients with previous ocular surgeries. Astigmatism was studied using the automatic keratometry of the same device. Keratometry with this system demonstrated high precision and repeatability and has been shown to produce better clinical results with toric IOLs than manual keratometry. [10]

### Statistical analysis

One eye was randomly chosen for each patient. The biometric data measured were entered into an Excel spreadsheet (Microsoft Office 2010; Microsoft, Redmond, WA). The statistical analysis was performed according to E9 guidelines of the ICH principles of statistics for clinical trials, using SPSS for Mac (version 21.0, Chicago, IL). The normality of the data was accessed with the Kolmogorov–Smirnov test. Since none of the studied variables had a normal distribution, nonparametric statistics were used. The Mann–Whitney U test was used for comparisons between groups. Correlations were performed using the Spearman coefficient. Regression models considering age, gender, LT, and CD were constructed to determine associations with the most relevant ocular biometric parameters (AL, ACD, and K). The results are expressed as the parameter mean value ± standard deviation (SD), and those with a value of p < .05 were considered statistically significant.

## Results

### Demographic data and biometric parameters

The demographic data and ocular biometric parameters of the patients are presented in Table 1.

**Table 1:** Demographic data and mean ocular biometric parameters in Portuguese population.

**Parameter**	**Mean ± SD (range)**		
Eyes (*n*)	6,506		
Patients (*n*)	6,506		
Age (years-old) Range	69 ± 10 (44–99)		
Females, *n* (%)	3,721 (57.2%)		
Right eyes, *n* (%)	1,678 (51.6%)		
	**Total**	**Males**	**Females**
Axial length (mm) ± SD Range	23.87 ± 1.55 (19.8–31.92)	23.99 ± 1.47 (20.03–31.92)	23.68 ± 1.46 (19.8–29.99)
Mean keratometry (D) ± SD Range	43.91 ± 1.71 (40.61–51.14)	43.46 ± 1.11 (40.93–51.14)	44.20 ± 1.29 (40.61–49.93)
Mean corneal astigmatism (D) ± SD Range	1.08 ± 0.84 (0–7.58)	1.09 ± 0.92 (0–7.58)	1.12 ± 0.86 (0–6.27)
Anterior chamber depth (mm) ± SD Range	3.25 ± 0.44 (2.04–5.28)	3.30 ± 0.40 (2.06–5.42)	3.14 ± 0.43 (2.04–4.99)
Lens thickness (mm) ± SD Range	4.32 ± 0.49 (2.73–5.77)	4.35 ± 0.49 (2.75–5.77)	4.38 ± 0.41 (2.73–5.42)
Corneal diameter (mm) ± SD Range	12.02 ± 0.46 (10.50–14.15)	12.03 ± 0.43 (10.51–14.15)	11.98 ± 0.49 (10.50–14.09)

**Fig 1:**
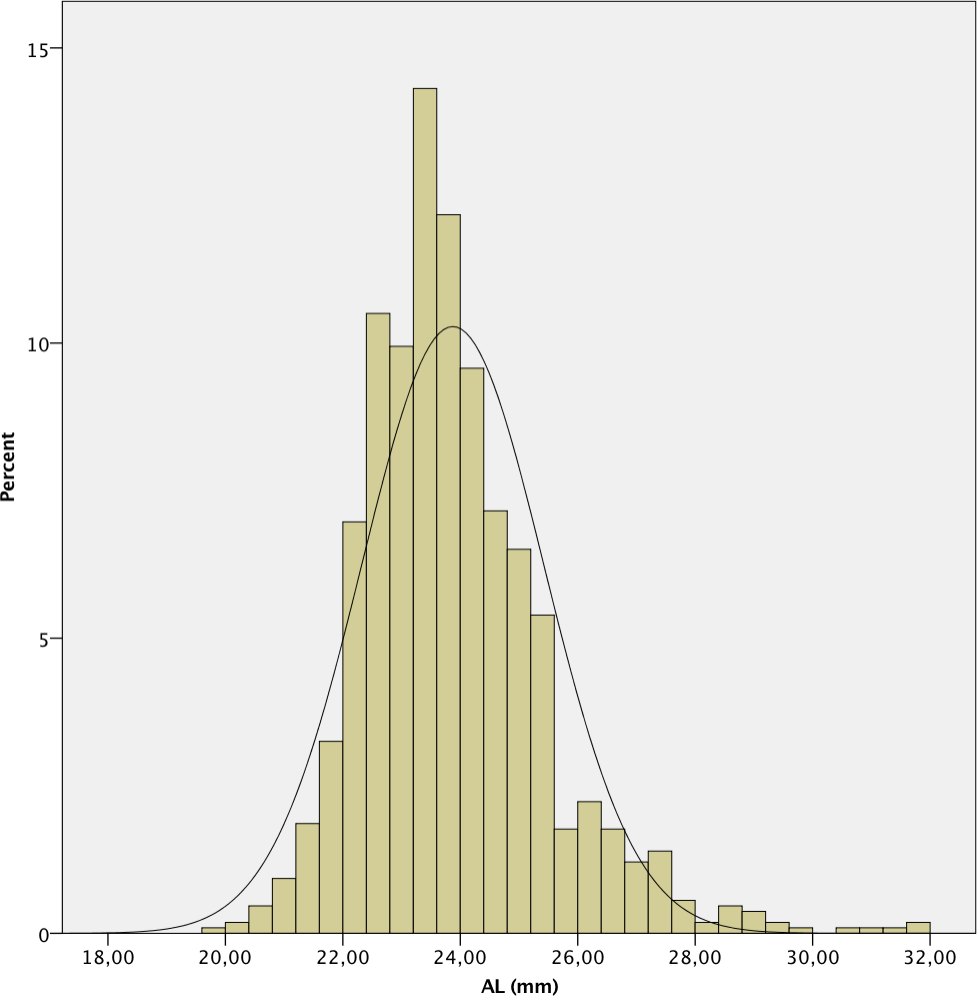
Histogram of axial length (AL) of the study population.

**Fig 2:**
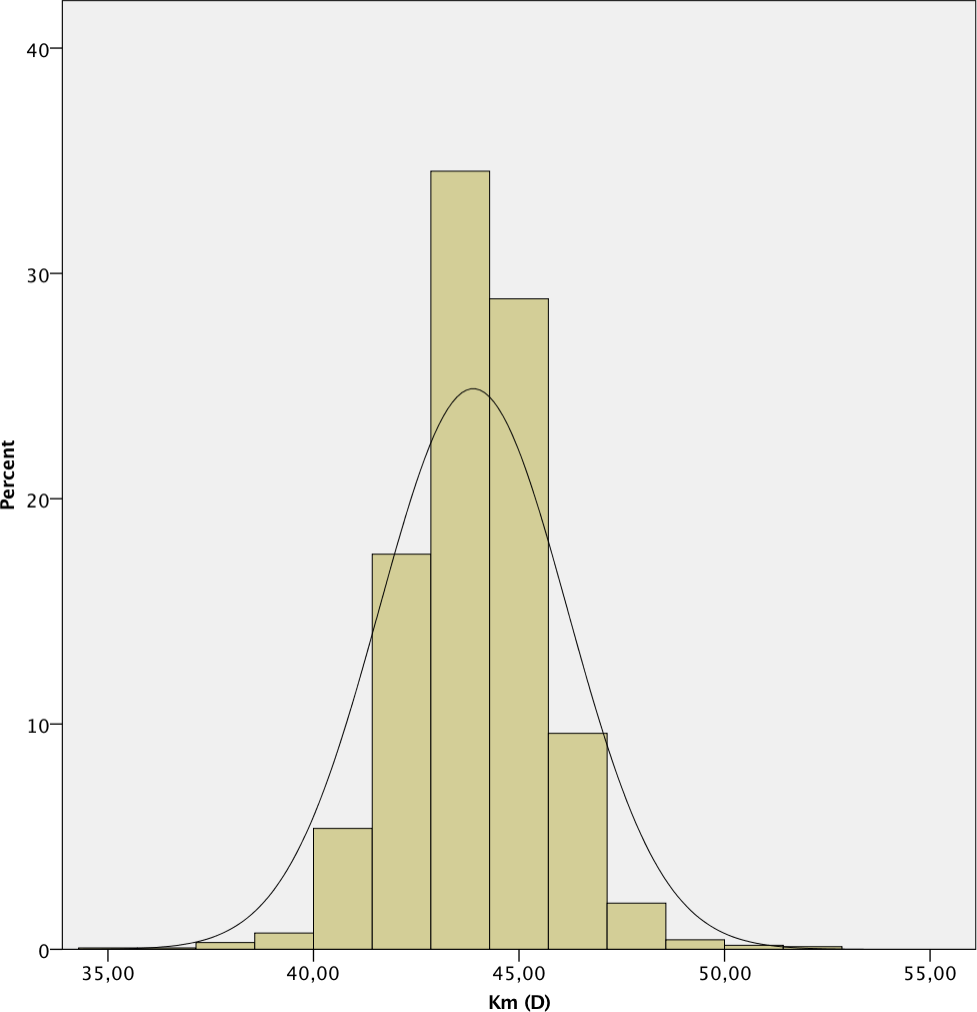
Histogram of mean keratometry (K) of the study population.

**Fig 3:**
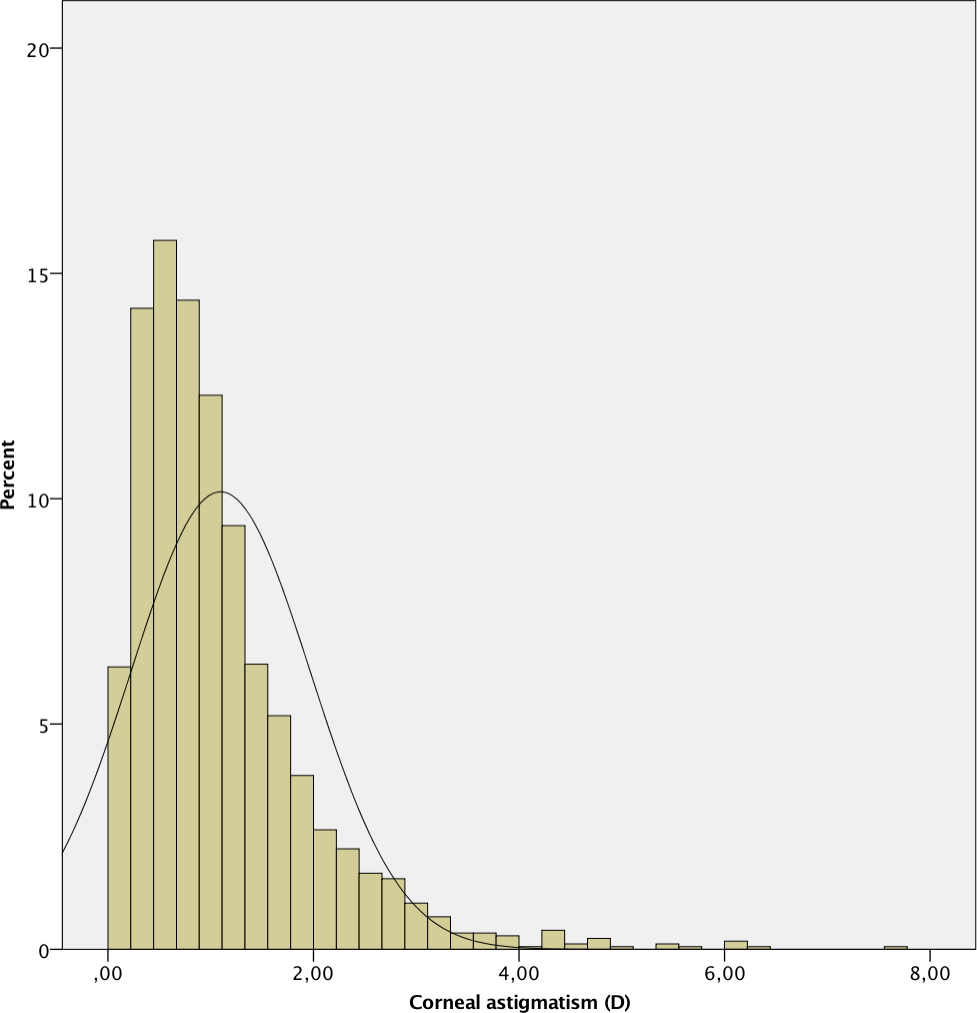
Histogram of corneal astigmatism of the study population.

**Fig 4:**
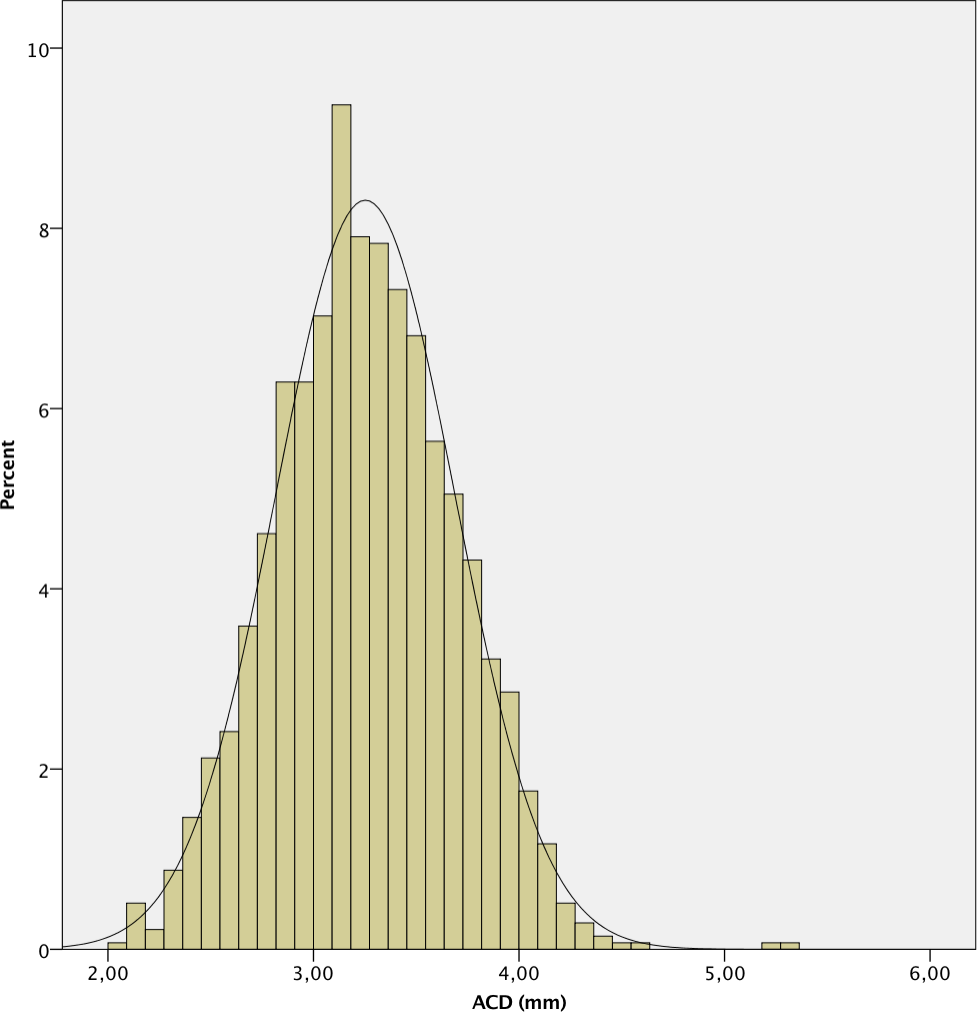
Histogram of anterior chamber depth (ACD) of the study population.

**Fig 5:**
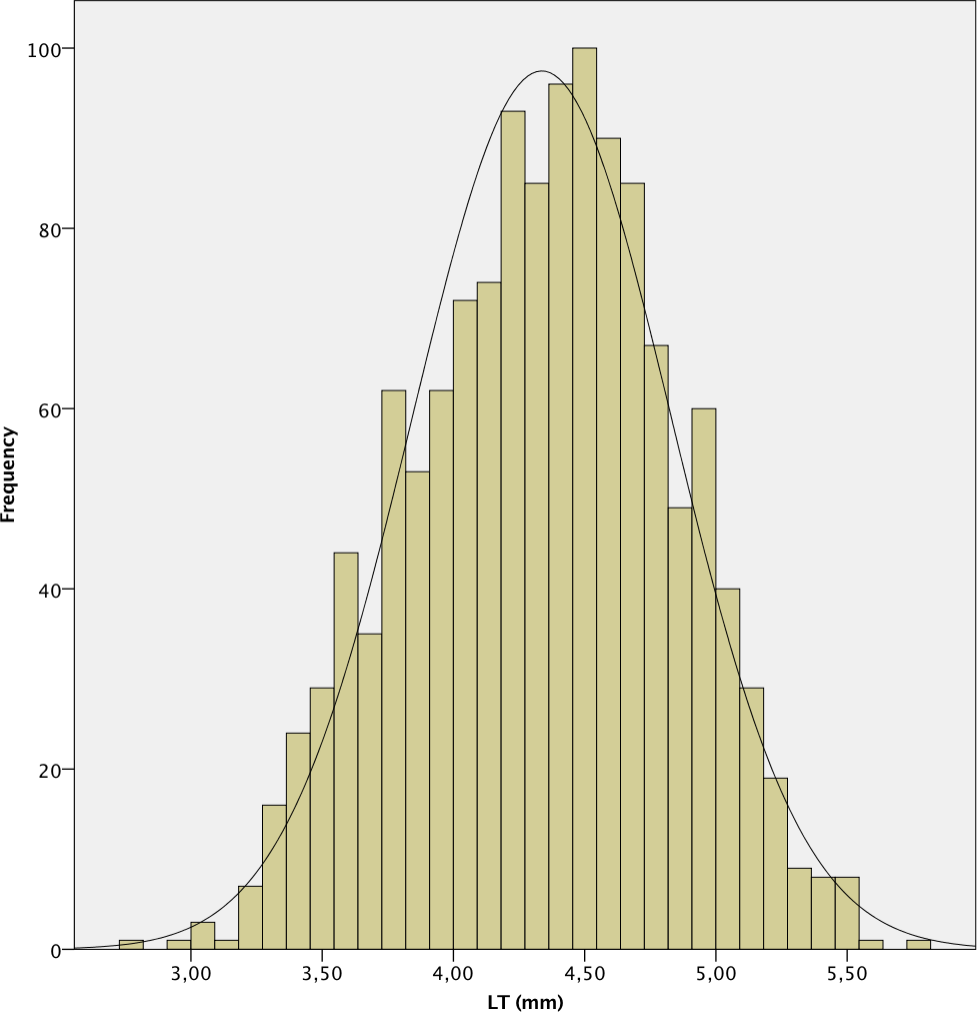
Histogram of lens thickness (LT) of the study population.

**Fig 6:**
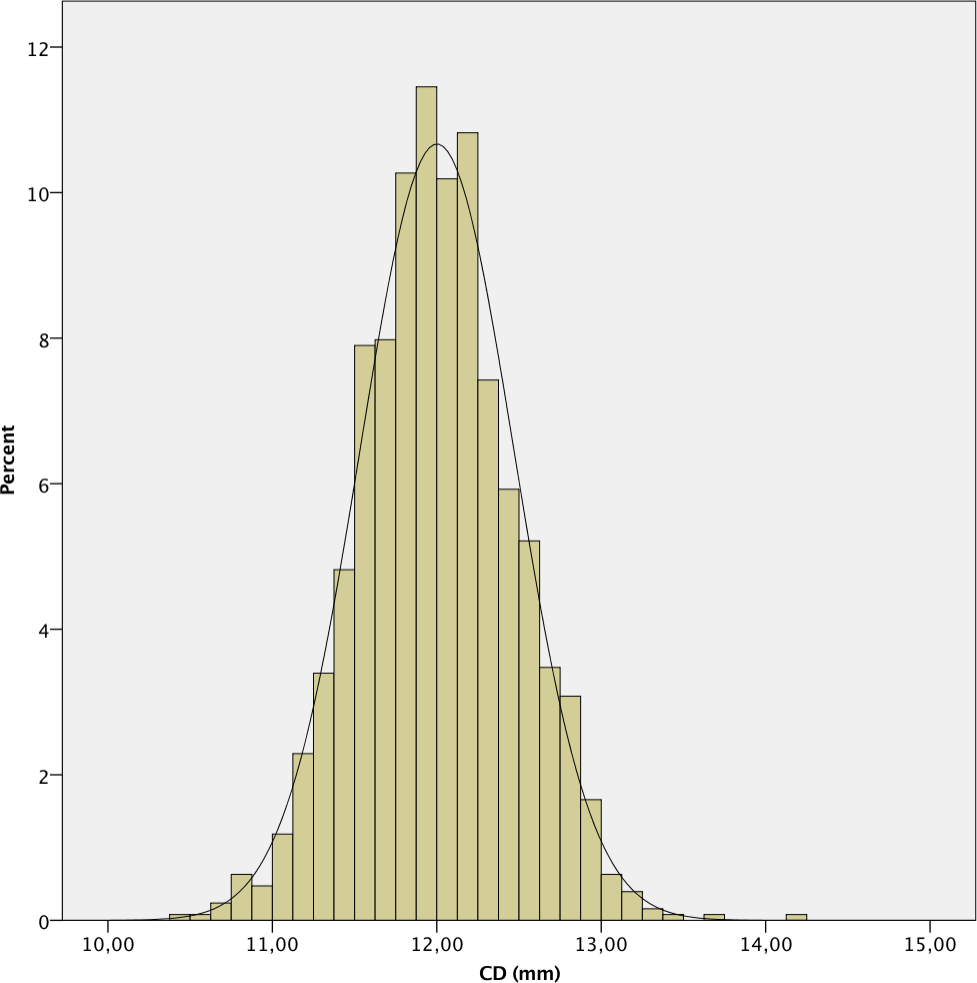
Histogram of corneal diameter (CD) of the study population.

The mean AL was 23.87 ± 1.55 mm. 241 (7.4%) eyes had an AL < 22.0 mm, 2,111 (64.9%) between 22.0 and 24.5 mm, 612 (18.8%) between 24.5 and 26.0 mm and 289 (8.9%) > 26.0 mm. A positive deviation and a leptokurtic distribution (kurtosis 2.804) were observed, with a significant deviation from normality, as in the other measured parameters (p < .001 in all cases except for CD, p = .049). The histograms of the distribution of the measured values of AL, K, ACD, LT and CD are shown in Figs 1–6.

Male eyes had longer ALs, deeper ACDs and flatter corneas than female eyes (p < .001); however, there was no statistically significant differences in the other parameters evaluated.

**Fig 7:**
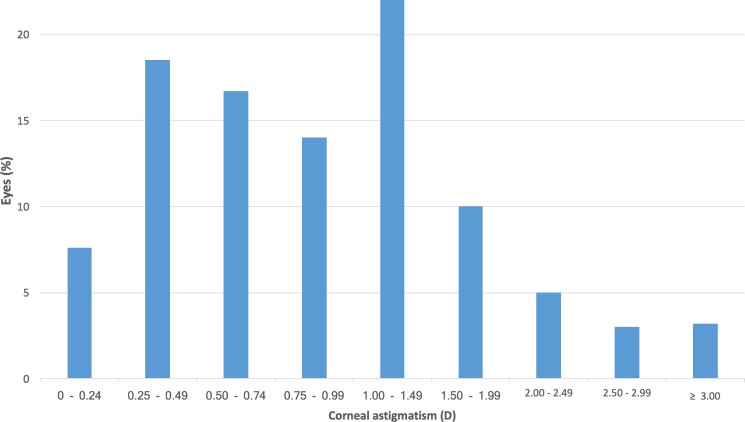
Distribution of corneal astigmatism in the study population.

The distribution of corneal astigmatism is shown in Fig 7. The mean corneal astigmatism was 1.08 ± 0.84 D (range 0–7.58), with 1415 (43.5%) eyes showing astigmatism ≥ 1 D. 1,513 (46.5%) eyes presented against-the-rule (ATR) astigmatism (steep meridian 0–30 degrees or 150–180 degrees), 1,077 (33.1%) eyes with-the-rule (WTR) (60 to 120 degrees), and 663 (20.4%) eyes were oblique (31–59 degrees or 121–149 degrees).

### Correlations

The AL, ACD, LT and CD were all significantly correlated between each other (p < .001). There was no significant correlation between age and any of the biometric parameters investigated (p > .05). The complete matrix of correlations is presented in Table 2.

**Table 2:** Matrix of correlations of ocular biometric parameters in Portuguese population.

			ACD	LT	CD	Mean K	Age	AL
Rho Spearman	ACD	Correlation coefficient	1,000	-.633**	.484**	-.045	-.018	.571**
		Sig.	.	< .001	< .001	.281	.500	< .001
	LT	Correlation coefficient	-.633**	1.000	-.245**	.032	-.001	-.334**
		Sig.	< .001	.	< .001	.256	.979	< .001
	CD	Correlation coefficient	.484**	-.245**	1.000	.048	-.009	.454**
		Sig.	< .001	< .001	.	.098	.761	< .001
	Mean K	Correlation coefficient	.040	.032	.048	1.000	-.035	.040
		Sig.	.148	.256	.098	.	.150	.150
	Age	Correlation coefficient	-.018	.012	-.009	-.035	1.000	-.007
		Sig.	.500	.443	.761	.150	.	.804
	AL	Correlation coefficient	.571**	-.334**	.454**	.040	-.007	1.000
		Sig.	< .001	< .001	< .001	.150	.804	.

**. Significant correlation (0.01).

Sig. = significance, ACD = anterior chamber depth (epithelium to lens), LT = lens thickness, CD = corneal diameter, Mean K = mean keratometry, AL = axial length.

### Regression models

Regression models were constructed for AL, ACD, and K considering age, gender, K, ACD, LT, and CD. A longer AL was associated with male gender (β = .082, p = .018), and deeper ACD (β = 0.512, p < .001), LT (β = .105, p = .007), and CD (β = .171, p < .001). A deeper ACD was associated with male gender (β = .571, p < .001), longer AL (β = .298, p < .001), wider CD (β = .253, p < .001), and thinner LT (β = − .496, p < .001). For K, there was a significant association with male gender (β = .313, p < .001) and no significant associations with the other studied parameters.

## Discussion

This study presents the normative values of the biometric parameters evaluated using optical biometry in a Portuguese population of candidates for cataract surgery. To our knowledge, this is the first study to characterize these parameters in this population.

### Mean values of biometric parameters

The mean values of the biometric parameters published in the different studies in the literature are described in Table 3.

**Table 3:** Mean values of biometric parameters published in previous studies.

Study	Country	Race	Measurement method	AL (mm)			ACD (mm)			Km (D)		
				Total	Males	Females	Total	Males	Females	Total	Males	Females
Ferreira et al.	Portugal	Caucasian	Lenstar	23.87	23.99	23.68	3.25	3.20	3.09	43.91	43.46	44.20
The Tanjong Pagar Survey [1]	Singapore	Chinese	US Contact	23.23	23.54	22.98	2.90	2.99	2.81	44.12	43.66	44.47
Cao et al. [14]	China	Chinese	US Contact	23.04	-	-	3.03	-	-	44.24	-	-
Los Angeles Latino Eye Study [2]	USA	Hispanic	US Contact	23.38	23.65	23.18	3.41	3.48	3.36	43.72	43.35	43.95
Hoffer [15]	USA	Caucasian	US Immersion	23.65	-	-	3.24	-	-	43.81	-	-
Jivrajka et al. [16]	USA	Caucasian	US Immersion	23.46	23.76	23.27	2.96	3.05	2.90	-	-	-
The Reykjavik Study [11]	Finland	Caucasian	US Contact	-	23.74	23.20	-	3.20	3.08	-	43.41	43.73
The Singapore Malay Eye Study [12]	Singapore	Malay	IOLMaster	23.55	-	-	3.10	-	-	44.12	-	-
The Blue Mountains Eye Study [13]	Australia	Caucasian	IOLMaster	23.44	23.75	23.20	3.10	3.16	3.06	43.42	43.01	43.74
The Beaver Dam Eye Study [6]	USA	Caucasian	IOLMaster	23.69	23.92	23.51	3.11	3.14	3.09	43.83	43.44	44.12
Hoffmann et al. [17]	Germany	Caucasian	IOLMaster	23.43	23.77	23.23	3.11	3.12	3.02	43.89	43.44	44.12
Knox Cartwright et al. [18]	United Kingdom	Caucasian	IOLMaster	23.40	23.76	23.20	-	-	-	43.90	43.45	44.18
Siahmed et al. [19]	France	Caucasian	IOLMaster	23.46	-	-	-	-	-	43.97	-	-
Olsen [20]	Denmark	Caucasian	IOLMaster	23.45	-	-	-	-	-	-	-	-

US = A-scan ultrasound biometry, AL = axial length, ACD = anterior chamber depth, Km = mean keratometry (values converted to D using the given refractive index)

In our study, we found that AL had a non-normal distribution with positive deviation and high kurtosis. This deviation and kurtosis are in accordance with the findings described in the Reykjavik Eye study, [11] Singapore Malay Eye study, [12] and the Blue Mountains Eye Study. [13]The normality of the distribution is variable in several studies. The mean AL in our study (23.87 ± 1.55 mm) is longer than the one reported in the Singapore and Chinese populations using applanation contact ultrasound. [5, 14] It is still slightly longer than that reported in the Hispanic population of Los Angeles and Singapore Malay [12] and in the studies of Hoffer [15] and Jivrajka et al [16] both in the USA and using immersion ultrasound. When compared with studies using optical biometry, the mean AL in our population is longer than that of the Caucasian population in Australia [13] and USA. [6] Using optical biometry, the mean AL in our study is longer than that published in several studies of European populations. [17–20] Although there may be differences explained by the AL measurement method, the AL in our study is longer than that published in the literature for different populations in studies using optical biometry, being closer to that reported in the Caucasian population of the USA [6, 15] than in European Caucasian populations. [17–20] In the latter populations, there is great similarity in the AL values reported in different countries; hence, in our study is longer than all of them, with a difference in mean values of about 0.40 mm. Although the majority of these studies used the IOLMaster 500 (Carl Zeiss AG, Jena, Germany) and our study used the Lenstar, this does not explain the differences found, since it was demonstrated by Hoffer et al. [9] that the IOLMaster and Lenstar AL biometry are not significantly different. The mean difference found is relevant, since a 1 mm error in AL results in a residual postoperative refractive error of 2.35 D in a 23.5 mm eye, 1.75 D in a 30.0 mm eye and 3.75 D in a 20 mm eye or about 2.0–4.0 D in the power of the implanted IOL. [21]

The mean keratometry in our study did not follow a normal distribution, with negative deviation and high kurtosis. These findings are similar to both the Singapore Malay Eye study [12] and the Blue Mountains Eye Study. [13] The mean keratometry in our study was 43.91 ± 1.71 D. This value is lower than that reported in the Chinese [2] and Singaporean [12] populations, being closer to those reported in the Caucasian population in Europe [18, 19] and USA. [15] Although the keratometry evaluation methods are different, there is a close relationship between the values reported in Caucasian populations, which are generally lower than in the Far Eastern populations. The difference observed with respect to Far Eastern populations is significant, representing a potential difference in the refractive error greater than 0.50 D. [21]

It is known that about 29 to 40% of patients undergoing cataract surgery have corneal astigmatism greater than 1 D, which is enough to prevent optimal visual acuity without optical correction. [22] In our series, the mean corneal astigmatism was 1.08 ± 0.84 D, with 43.5% of the eyes showing astigmatism ≥ 1 D. These values are higher than those reported in most studies, such as those by Ferrer-Blasco et al. [22] (34.8%) in Spain and by Hoffmann et al. [17] in Germany (36%). It is known that corneal astigmatism varies significantly with age, increasing the prevalence of ATR astigmatism. [23] In our study, the majority of eyes (46.5%) had ATR astigmatism, which is in agreement with the age range of the evaluated population, with a mean of 69 years-old.

The mean ACD in our population (3.25 ± 0.44 mm) was higher than that reported in most studies in Eastern [2, 12, 14] and in Western populations [6, 11, 13, 17], and it is comparable with that reported by Hoffer in the USA [15]. The differences found may be partly because of the measurement method used, since Lenstar uses laser optical biometry to measure ACD, while the IOLMaster 500, uses an optical slit image. Hoffer et al [9] have reported that ACD values with Lenstar are higher than those measured with IOLMaster 500.

In our series, the mean LT was 4.32 ± 0.49 mm, and it was directly proportional to age and inversely proportional to AL. These findings confirm those of the studies by Jivrajka et al. [16] and Hoffer [15, 24], although LT in our study was thinner than those studies reported.

The mean CD in our study (12.02 ± 0.46 mm) was similar to that reported in other series in the literature. [13, 17]

### Relationship with gender and age

In this study, male eyes had longer ALs, deeper ACDs and flatter corneas than female patients did, and a gender difference with respect to the other investigated parameters was not statistically significant. These results are in accordance with those in the literature, especially in populations from Germany, [17] Australia, [13] USA, [6, 16] and Iceland. [11] It is interesting to note that in a paper to be published, Hoffer et al. found a constant 0.50 mm difference in AL between genders, much higher than the 0.31 mm we found in this study. According to the Beaver Dam Eye Study [6] the height adjustment of individuals can explain all the differences found between the genders, however other studies have adjusted for height and weight and found that the differences still existed. Since gender and race appear to be important determinants of ocular biometric parameters, it may be important to consider them in the calculation of the IOL for cataract surgery, as shown by the appearance of the first 5th-generation formula, the Hoffer-H-5, which uses the same basic structure as the Holladay 2 formula but considers gender and ethnicity to reduce the error associated with the use of generalized population regression factors.

In contrast with most studies, [13, 17] there was no significant correlation between age and AL, ACD, or K. In the study by Hoffmann et al., [17] the results were similar to those observed in our series, and no correlation was found between age and AL. The interpretation of these differences is complex and would require adjustments for the refraction, height, age, and even educational level of the studied population.

### Correlations between parameters

In this series, there was a positive correlation between AL and ACD, and K (not statistically significant) and CD, and a negative correlation between AL and LT. These results are in agreement with those reported in the literature [13, 15, 16] except for K, whose correlation with AL is inverse in most series, showing the emmetropic relationship between AL and corneal curvature. [15] Although there may be population differences and the correlation with refractive error has not been addressed in this study, the different published studies in the literature reported keratometry evaluated with manual, automatic, or IOLMaster keratometry, and these values cannot be directly compared with ours because of the different methods of measurement and refractive indices used.

### Regression models

In regression models for AL, ACD, and K considering age, gender, K, ACD, LT, and CD, the major determinants of AL were ACD and CD, and gender was not significant. Unlike the finding in other series, age was not a significant determinant. [6, 12] An association between ACD and CD and LT was also observed.

In conclusion, the present study presents normative biometric parameters and their relationships in a Portuguese population. These results may be relevant not only in the evaluation of the refractive error but also in the IOL calculation for cataract surgery. The obtained AL, ACD, and mean K values were closer to the US population than most published series in different European Caucasian populations, and the disparities found could represent differences greater than 1 D in both the refractive error and the IOL power. Corneal astigmatism in the present study was higher than that in most published series, which may affect the type of IOL to be implanted.
